# Diagnostic delays and persistent symptoms in lymphogranuloma venereum among men who have sex with men in Poland: a single-centre case series

**DOI:** 10.1186/s12879-026-13354-y

**Published:** 2026-05-18

**Authors:** Damian Kadylak, Justyna Czarny-Kamm, Małgorzata Sokołowska-Wojdyło

**Affiliations:** 1 Department of Dermatology, Venereology and Allergology, Faculty of Medicine, Medical University of Gdańsk, Smoluchowskiego 17 Street, Gdańsk, 80-214 Poland; 2 Department of Dermatology, Venereology and Allergology, University Clinical Centre, Smoluchowskiego 17 Street, Gdańsk, 80-214 Poland

**Keywords:** Lymphogranuloma venereum, *Chlamydia trachomatis*, MSM, Poland, Sexually transmitted infections, Proctitis

## Abstract

**Background:**

Lymphogranuloma venereum (LGV) caused by *Chlamydia trachomatis* serovars L1–L3 is increasingly reported among men who have sex with men (MSM) in Western Europe, yet appears to remain underrecognised in Central and Eastern Europe. We describe clinical characteristics, diagnostic pathways, and outcomes of LGV cases in Poland to highlight recognition barriers and public health implications.

**Methods:**

We conducted a retrospective case series of symptomatic MSM diagnosed with LGV at a dermatovenereology outpatient clinic in Gdańsk, Poland, between 1 December 2023 and 31 October 2025. Cases were confirmed by *C. trachomatis* nucleic acid amplification test followed by LGV-discriminatory PCR. We extracted demographic, clinical, laboratory, and treatment data from electronic records.

**Results:**

Seven MSM (median age 28 years, range 25–68) were diagnosed with LGV; five (71%) were living with HIV. Common symptoms included rectal bleeding, rectal pain, and mucopurulent discharge (each 71%). Median time from symptom onset to diagnosis was 6 months (range 2 weeks–7 months); patients consulted a median of 3 physicians (range 1–5) before diagnosis. Five underwent colonoscopy with findings initially suggesting inflammatory bowel disease or malignancy. Concurrent sexually transmitted infections were frequent, including gonorrhoea (57%), syphilis (29%), and herpes simplex virus (29%). All received doxycycline 100 mg twice daily for 21 days. Three patients—all with diagnostic delays ≥ 6 months—reported persistent anorectal symptoms post-treatment despite microbiological cure.

**Conclusions:**

LGV may be underdiagnosed in Poland, and diagnostic delays exceeding six months may be associated with persistent symptoms despite microbiological cure. Enhanced clinician awareness, routine sexual history-taking in both primary and specialist care, and improved access to LGV-discriminatory testing are needed to reduce morbidity and prevent irreversible complications in this vulnerable population.

## Background

Lymphogranuloma venereum (LGV), caused by invasive *Chlamydia trachomatis* serovars L1–L3, manifests as either classical inguinal lymphadenopathy with genital ulceration (‘buboes’) or — now predominant among men who have sex with men (MSM) — anorectal proctitis with mucopurulent discharge, bleeding, and pain that often mimics inflammatory bowel disease (IBD) or malignancy [1–3]. Because rectal LGV rarely produces external lesions, diagnosis is frequently delayed [3].

At the European level, population-based incidence or prevalence estimates for LGV are not available, as surveillance systems differ substantially between countries and many do not generate representative denominator data [4]. Nevertheless, surveillance reports provide important insight into the burden and distribution of diagnosed LGV cases. Notably, much of the available epidemiological data on LGV in Europe originates from Western European countries.

According to the European Centre for Disease Prevention and Control (ECDC), 22 EU/EEA countries reported 3,075 confirmed LGV cases in 2023. This represents a 41% increase compared with 2022 [4]. Nearly all reported cases occurred among MSM, and among cases with known HIV status, approximately 38% were reported in individuals living with HIV [4]. Case notifications were highly uneven across Europe: two countries (Spain and the Netherlands) accounted for 77% of all reported cases, while several countries reported none [4].

Across Europe, LGV is well-established among MSM but remains underrecognised, particularly in settings with limited genotyping capacity [2, 5]. In addition, studies based on systematic LGV testing of rectal *C. trachomatis*-positive samples among MSM suggest that LGV represents a substantial proportion of diagnosed rectal chlamydial infections. A multicentre European study found LGV in 25.6% of rectal *C. trachomatis*-positive specimens from MSM, with country-specific proportions ranging from 14.4% in the UK to 47.6% in Austria [2].

This western European epidemiological pattern contrasts sharply with the situation in Poland. Only five confirmed LGV cases were reported between 2013 and 2024 [6]. This near-absence of reports, despite widespread European transmission, suggests potential underrecognition rather than true rarity.

We describe seven symptomatic LGV cases diagnosed between December 2023 and October 2025 at a dermatovenereology outpatient clinic in Gdańsk, highlighting diagnostic challenges, treatment outcomes, and implications for clinical practice and surveillance.

## Methods

### Study design and setting

We conducted a retrospective case series at the outpatient clinic Department of Dermatology, Venereology and Allergology, University Clinical Centre in Gdańsk, a tertiary urban referral centre providing specialised sexually transmitted infection (STI) care. The clinic serves approximately 1,000 visits annually for STI diagnosis and treatment. The study period covered the period from 1 December 2023 to 31 October 2025.

### Case definition and identification

Cases were defined as patients with symptomatic LGV infection identified through routine clinical practice and laboratory surveillance. LGV testing was performed in patients presenting with anorectal or inguinal symptoms suggestive of LGV, or during diagnostic evaluation for other sexually transmitted infections when rectal *C. trachomatis* was detected. LGV testing was not routinely offered for either routine or targeted screening in this setting. All cases had rectal or inguinal specimens positive for *C. trachomatis* by nucleic acid amplification test (NAAT), with subsequent confirmation of L1, L2, or L3 serovars by discriminatory polymerase chain reaction (PCR).

### Laboratory methods

Specimens collected from affected anatomical sites were tested for *C. trachomatis* using nucleic acid amplification tests (NAATs). LGV infection was confirmed using LGV-discriminatory PCR differentiating L serovars (L1–L3) from non-LGV serovars.

Testing for concurrent sexually transmitted infections was performed as part of routine syndromic evaluation and included NAATs from rectal, pharyngeal, and urine specimens for *C. trachomatis*,* Neisseria gonorrhoeae* and herpes simplex virus-1/2 (HSV-1/2). Serological testing for syphilis, HIV, and hepatitis C virus was performed as part of routine clinical evaluation, with HIV testing omitted in patients with a known prior diagnosis.

### Data collection

We retrospectively reviewed electronic medical records and extracted data on demographics (age, HIV status), clinical presentation, diagnostic pathway, procedures performed, concurrent STIs, treatment, and outcomes. Electronic records were limited to encounters within our institution; information on prior consultations at other healthcare facilities was based on patient self-report as documented in the medical record. Time to diagnosis was defined as the interval between symptom onset and laboratory confirmation.

### Treatment and follow-up

All patients received doxycycline 100 mg orally twice daily for 21 days in accordance with European guidelines [1]. Test-of-cure was performed selectively in patients with incomplete clinical response or persistent symptoms after completion of therapy.

### Statistical analysis

Descriptive statistics were used to summarise patient characteristics. Continuous variables are presented as median and range, and categorical variables as counts and percentages. Analyses were performed using standard descriptive methods.

## Results

### Patient characteristics

We identified seven MSM diagnosed with LGV during the study period (Table 1). Median age was 28 years (range 25–68). Five patients (71%) were living with HIV, including one newly diagnosed concurrently with LGV.

**Table 1:** Clinical characteristics and diagnostic course of seven patients with lymphogranuloma venereum

Case	Age (years)	HIV status	Time to diagnosis (months)	No. physicians consulted	Initial suspected diagnosis	Main symptoms	Co-infections	Key diagnostic findings (colonoscopy / histopathology / imaging)	Outcome* / follow-up
1	68	HIV+ (on ART)	6	4	Colorectal cancer	Rectal bleeding, mucopurulent rectal discharge, rectal pain, constipation	Syphilis; HSV-1/2 (anal)	- Three colonoscopies: inflamed rectal mucosa. - Multiple biopsies: chronic proctitis without dysplasia or malignancy.	Persistent anorectal discomfort and mild fecal incontinence; follow-up pelvic MRI: chronic post-inflammatory rectal wall changes despite negative LGV PCR at test-of-cure
2	26	HIV+ (on ART)	6	3	Inflammatory bowel disease	Rectal bleeding, mucopurulent rectal discharge, rectal pain, diarrhea, abdominal pain	*N. gonorrhoeae* (rectal)	- Colonoscopy: deep 8–10 cm rectal ulcer and 2–3 cm sigmoid ulcer. - Biopsy: chronic active inflammation with granuloma formation; no dysplasia or malignancy.	Persistent anorectal discomfort despite negative LGV PCR at test-of-cure
3	38	HIV+ (on ART)	4	3	Colorectal cancer	Rectal bleeding, mucopurulent rectal discharge, rectal pain	*N. gonorrhoeae* (rectal, pharyngeal)	- Colonoscopy: anal canal ulceration > 50% circumference. - Biopsy: chronic active inflammation with ulcer base granulation tissue; no dysplasia or malignancy. - Pelvic MRI: rectal wall thickening with inflammatory changes.	Complete resolution
4	28	HIV+ (new diagnosis)	7	5	Inflammatory bowel disease vs. infection	Rectal bleeding, rectal pain, constipation, diarrhea	Syphilis	- Colonoscopy: rectosigmoid erosions (5–10 mm) with fibrin and oedema. - Biopsy: focal active colitis without dysplasia or malignancy.	Complete resolution
5	25	HIV–	0.5	2	STI	Mucopurulent rectal discharge	None detected	No endoscopy or imaging performed.	Complete resolution
6	28	HIV+ (on ART)	1	1	LGV (clinically suspected)	Unilateral inguinal lymphadenopathy, urethral discharge	*N. gonorrhoeae* (rectal)	Ultrasound: multiple enlarged inguinal nodes 12–40 mm.	Complete resolution
7	45	HIV–	6	4	Inflammatory bowel disease vs. colorectal cancer	Rectal bleeding, mucopurulent rectal discharge, rectal pain	*N. gonorrhoeae* (rectal); HSV-1/2 (anal)	- Colonoscopy: performed; detailed report not available in medical record. - Biopsy: nonspecific granulomatous inflammation and plasma-cell infiltrate; no dysplasia or malignancy	Persistent anorectal discomfort despite negative LGV PCR at test-of-cure

* after completing a 21-day course of doxycycline 100 mg twice daily

Abbreviations: HIV, human immunodeficiency virus; ART, antiretroviral therapy; HSV, herpes simplex virus; LGV, lymphogranuloma venereum; STI, sexually transmitted infection; MRI, magnetic resonance imaging; PCR, polymerase chain reaction

### Clinical presentation and diagnostic pathway

Six patients presented with anorectal symptoms and one with inguinal lymphadenopathy. The most frequent symptoms were rectal bleeding (5/7, 71%), rectal pain (5/7, 71%), and mucopurulent rectal discharge (5/7, 71%). Patients consulted a median of three physicians (range 1–5) before the correct diagnosis was established, most commonly family doctors, gastroenterologists, surgeons, or oncologists. Median time from symptom onset to laboratory confirmation was six months (range 2 weeks–7 months).

Five patients underwent colonoscopy — some more than once — and all five had endoscopic findings initially interpreted as suggestive of IBD or colorectal carcinoma. Two patients were referred through urgent oncological diagnostic pathways before LGV was considered. Histopathological examination revealed chronic inflammatory changes of variable intensity among the five patients who underwent biopsy: three (60%) showed non-specific active proctitis, whereas two (40%) demonstrated granulomatous inflammation with microabscess formation. No dysplasia or neoplastic features were detected.

### Microbiological findings and co-infections

All rectal samples tested positive for *C. trachomatis* L1–L3 by discriminatory PCR. Six patients (86%) had at least one concurrent STI: *N. gonorrhoeae* (4/7, 57%), syphilis (2/7, 29%), and HSV-1/2 (2/7, 29%).

### Treatment and outcomes

All patients received doxycycline 100 mg twice daily for 21 days. Four patients (57%) achieved complete clinical and microbiological resolution. Three patients (43%) —all with diagnostic delays ≥ 6 months—reported persistent anorectal symptoms after treatment, including chronic discomfort and mild fecal incontinence in one case. In these symptomatic patients, test-of-cure PCR performed after completion of therapy was negative for LGV, suggesting that ongoing symptoms were more consistent with post-inflammatory sequelae than with persistent infection.

In one patient with persistent fecal incontinence, pelvic magnetic resonance imaging was performed due to ongoing symptoms. The examination demonstrated circumferential rectal wall thickening over a 6 cm segment above the anal sphincters, with associated inflammatory changes in the mesorectal fat and small reactive mesorectal lymph nodes. No diffusion restriction suggestive of malignancy was observed. Overall, these findings were interpreted as chronic post-inflammatory changes rather than active infection or neoplasia.

## Discussion

In this single-centre retrospective case series, we describe the clinical presentation, diagnostic pathways, and outcomes of seven MSM diagnosed with lymphogranuloma venereum in Poland. Our findings highlight prolonged diagnostic delays, frequent misclassification as non-infectious disease, and the potential clinical consequences of delayed recognition in a setting with limited surveillance and genotyping capacity.

### Diagnostic delay and its causes

In this series, patients consulted a median of three physicians over several months before a correct diagnosis was established, reflecting important gaps in clinical recognition of LGV. Diagnostic pathways were often prolonged and involved extensive invasive investigations, including repeated colonoscopies and oncological referral, before an infectious aetiology was considered.

These prolonged diagnostic pathways were compounded by the clinical and histopathological features of LGV, which frequently mimicked non-infectious disease. Granulomatous inflammation was identified in two cases in our series, a histopathological feature that may mislead clinicians toward a diagnosis of Crohn’s disease and reinforce initial non-infectious diagnostic pathways. Similarly, rectal bleeding and mass-like inflammatory changes prompted oncological evaluation in some patients, with LGV considered only after malignancy had been excluded.

Beyond clinical mimicry, diagnostic delay at the individual patient level reflects broader structural limitations, including low clinical awareness and restricted access to LGV-specific testing. As outlined in the Background, existing surveillance data likely underestimate the true burden of disease [4], and limited awareness of LGV epidemiology may contribute to its omission from differential diagnosis. Failure to consider STIs in the diagnostic work-up can lead to delayed or inappropriate management; a properly conducted sexual history remains essential for diagnosing LGV in patients presenting with anorectal complaints [3]. However, heteronormative communication and insufficient training in sexual diversity may discourage disclosure of same-sex behaviour, resulting in incomplete histories and misdiagnosis [7].

The diagnostic challenge is further contextualized by the epidemiological profile of LGV in Europe. The high proportion of patients living with HIV in this series (71%) aligns with European data indicating that LGV transmission occurs predominantly within MSM networks with high HIV prevalence. Recent multicentre studies report HIV co-infection rates between 50% and 80% among LGV cases across Europe, with national surveillance data from France showing 72% of cases among MSM living with HIV [1, 2, 5]. This overlap likely reflects shared behavioural and network-level risk factors, underscoring the importance of integrated STI and HIV screening in MSM presenting with proctitis and highlighting that clinicians working in HIV care settings should maintain high clinical suspicion for LGV.

### Clinical consequences of delayed diagnosis

Rather than reiterating individual clinical outcomes, our findings suggest that prolonged, untreated LGV-related inflammation may predispose to persistent anorectal dysfunction even after microbiological cure. LGV is an invasive infection involving lymphatic tissue, and delayed treatment can result in sustained inflammatory injury with subsequent lymphatic obstruction, tissue remodeling, and functional impairment [3].

In this context, the persistent anorectal symptoms observed in all three patients with diagnostic delays of six months are biologically plausible and consistent with post-inflammatory sequelae rather than ongoing infection. Imaging findings available for one patient with persistent fecal incontinence further support this interpretation, demonstrating rectal wall thickening and mesorectal inflammatory changes without features of active infection or malignancy.

Although no strictures or fibrosis were documented on follow-up examination, the clinical course observed in these patients underscores that delayed recognition of LGV may lead to long-term functional consequences, even when appropriate antibiotic therapy is eventually administered and microbiological clearance is achieved.

### Surveillance and diagnostic gaps in Poland

LGV surveillance in Poland remains limited. As described in the Background, only five confirmed cases were reported nationally between 2013 and 2024, despite a marked increase in reported cases across the EU/EEA [4, 6]. Given the absence of population-based incidence data and the uneven distribution of reported cases across Europe, this discrepancy suggests potential underrecognition rather than true absence of disease.

In Poland, most diagnostic facilities perform standard *C. trachomatis* NAATs without L-serovar differentiation. As a result, LGV infections may remain unrecognised or unreported, particularly when rectal symptoms are attributed to non-infectious conditions such as inflammatory bowel disease or malignancy. By comparison, countries with established STI surveillance frameworks and broader access to genotyping consistently report substantially higher case numbers; in 2023, Spain and the Netherlands reported 1,806 and 577 LGV cases, respectively [4]. In several Western European settings, diagnostic algorithms incorporate routine or reflex LGV genotyping of rectal *C. trachomatis*-positive specimens among MSM, supported by national reference laboratories and integrated STI surveillance systems [1, 2, 5]. Such approaches may provide a useful framework for improving LGV detection, reporting, and surveillance in Poland.

### Clinical and public health implications

Delayed recognition of LGV can lead to prolonged morbidity and unnecessary invasive investigations. In our series, multiple colonoscopies and urgent oncological referrals were performed before LGV was considered, illustrating the potential clinical and economic consequences of misdiagnosis. High rates of concurrent STIs further complicate clinical assessment and support the need for comprehensive syndromic STI testing in MSM presenting with proctitis.

Improving clinician awareness and education, normalising sexual history-taking in both primary and specialist care, and expanding access to LGV-discriminatory molecular testing are likely to facilitate earlier recognition. In settings where diagnostic delays are common, early empirical treatment for LGV may be considered in selected high-risk patients while awaiting confirmatory testing [1]. At the health system level, strategies to improve LGV detection could include reflex LGV genotyping of rectal *C. trachomatis*–positive samples in high-risk populations and expansion of laboratory capacity for discriminatory PCR.

Further research is needed to better define the clinical and economic impact of delayed diagnosis. Studies incorporating structured clinical follow-up and functional assessment (such as anorectal manometry) would help clarify long-term outcomes, while health economic analyses could assess the cost-effectiveness of expanded LGV testing strategies.

### Limitations

This study has several limitations. As a single-centre retrospective case series, it cannot estimate population-level LGV incidence or prevalence. Cases were identified through symptomatic presentation, potentially introducing selection bias toward more severe disease. Data on the total number of patients tested for LGV were unavailable, precluding assessment of diagnostic yield. Retrospective data collection and reliance on patient self-report for prior healthcare encounters may also have introduced recall bias and information bias. The absence of a comparison group limits our ability to establish a causal relationship between diagnostic delay and persistent symptoms. In addition, detailed behavioural risk factors (such as number of sexual partners or chemsex practices) could not be systematically assessed due to the retrospective design, limiting epidemiological contextualisation. Future multi-centre studies incorporating systematic LGV genotyping and denominator data on rectal *C. trachomatis* testing would allow more accurate estimation of LGV prevalence and diagnostic coverage in Poland.

## Conclusions

In the absence of population-based incidence data, our findings suggest that LGV may be underrecognised in Poland, with prolonged diagnostic delays potentially associated with persistent anorectal symptoms despite microbiological cure. Beyond prolonged symptom duration, delayed recognition may contribute to post-inflammatory structural and functional anorectal changes, as illustrated by persistent dysfunction in patients with delayed diagnosis.

Enhanced clinician awareness, routine sexual history-taking, and improved access to LGV-discriminatory PCR are needed to support earlier diagnosis. Strengthening national surveillance through systematic genotyping of rectal *C. trachomatis* infections could improve case detection and better align Poland with European diagnostic and reporting standards.

## Data Availability

Data sharing is not applicable to this article as no datasets were generated during the current study.

## Abbreviations

ART: Antiretroviral therapy
HIV: Human immunodeficiency virus
HSV: Herpes simplex virus
IBD: Inflammatory bowel disease
LGV: Lymphogranuloma venereum
MSM: Men who have sex with men
NAAT: Nucleic acid amplification test
PCR: Polymerase chain reaction
STI: Sexually transmitted infection
